# The Discrimination Ratio derived from Novel Object Recognition tasks as a Measure of Recognition Memory Sensitivity, not Bias

**DOI:** 10.1038/s41598-018-30030-7

**Published:** 2018-08-01

**Authors:** Magali H. Sivakumaran, Andrew K. Mackenzie, Imogen R. Callan, James A. Ainge, Akira R. O’Connor

**Affiliations:** 1 University of St Andrews, School of Psychology and Neuroscience, St Andrews, KY16 8JP United Kingdom; 2 Nottingham Trent University, School of Social Sciences, Nottingham, NG1 4FQ United Kingdom

## Abstract

Translational recognition memory research makes frequent use of the Novel Object Recognition (NOR) paradigm in which animals are simultaneously presented with one new and one old object. The preferential exploration of the new as compared to the old object produces a metric, the Discrimination Ratio (*DR*), assumed to represent recognition memory sensitivity. Human recognition memory studies typically assess performance using signal detection theory derived measures; sensitivity (*d*′) and bias (*c*). How *DR* relates to *d*′ and *c* and whether they measure the same underlying cognitive mechanism is, however, unknown. We investigated the correspondence between *DR* (eye-tracking-determined), *d*′ and *c* in a sample of 37 humans. We used dwell times during a visual paired comparison task (analogous to the NOR) to determine *DR*, and a separate single item recognition task to derive estimates of response sensitivity and bias. *DR* was found to be significantly positively correlated to sensitivity but not bias. Our findings confirm that *DR* corresponds to *d*′, the primary measure of recognition memory sensitivity in humans, and appears not to reflect bias. These findings are the first of their kind to suggest that animal researchers should be confident in interpreting the *DR* as an analogue of recognition memory sensitivity.

## Introduction

Comparative studies of recognition memory have been dominated by the use of the (spontaneous) novel object recognition behavioural paradigm (referred to hereafter as the NOR task). Since the development of the NOR task^1^, the original paper has received more than 1,590 citations (Web of Science Core Collection, retrieved October 31, 2017), and an informal Google Scholar search for “novel object recognition” returns more than 3 million results. It has been estimated that approximately 43,000 rats and mice have been used in NOR experiments published in peer-reviewed journals between 2008 and 2012^2^. Its widespread usage likely reflects the relative ease in administering the task. It requires no rule learning or training, exploiting instead rodents’ innate predisposition to preferentially orient towards, and explore, new rather than old objects^3^. This innate bias in animals’ exploratory behaviour is thought to reflect underlying memory discrimination or sensitivity. Consequently, it is assumed that NOR performance is a valid indicator of how well an animal is able to discriminate an old stimulus from a new stimulus.

NOR tasks have a familiarisation sample phase and a test phase. To establish initial familiarity, an animal is presented with, and explores, two identical copies of an object (object A) in the test environment. Following a delay, the animal is next presented with the test component comprising the presentation of a new copy of object A and a novel object B in the same environment. If the animal orients preferentially to object B, an inference is made that the animal has “recognised” object A, thereby showing a sensitivity to discriminate old from new. Conversely, if the animal orients to and explores both objects equally, it is assumed that the animal demonstrates insufficient memory sensitivity to perform the recognition discrimination^4^. NOR task performance is quantified using the Discrimination Ratio (*DR*)^1^. The *DR* reflects the duration of exploration for the new (*T*_*new*_) compared to the old object (*T*_*old*_) as a proportion of the animal’s total exploration time (*T*_*total*_*;* see Eq. 1), and is thus equivalent to the d2 measure first developed when the NOR was introduced^1^. Despite the heavy reliance on the NOR task in the comparative literature, and the assumptions inherent in using it as a measure of recognition memory, we have little understanding of how the *DR* relates to the dissociable components of sensitivity and bias obtained from recognition memory judgement tasks in the human literature. This is addressed in the current experiment by assessing the correlations between the *DR* and measures of sensitivity and bias.

1 $$DR=\,\frac{{T}_{(new)}-\,{T}_{(old)}}{{T}_{(total)}}$$

Sensitivity and bias are useful and widely used concepts which can be quantified in humans using traditional, single item recognition memory tasks (referred hereafter as SIR tasks)^5^. Sensitivity—parameterised within signal detection theory as discriminability (*d*′)—refers to the ability to accurately differentiate old from new items. The better an individual is able to correctly identify old items as “old” (Hits; *Hs*), and new items as “new” (Correct Rejections; *CRs*), the greater their *d*′. Sensitivity thus considers both *H* and *CR* rates (see Eq. 2; False Alarms [*FAs*] are obtained by calculating the error rate for old items—the equivalent of 1-CR rate; equations use standardised z-scores of these, denoted by z). However, there is typically a set of items that fall within a mnemonically ambiguous range, such that their classification as either “old” or “new” is unclear. Under such circumstances, bias—parameterised within signal detection theory as criterion placement (*c*)—quantifies the tendency to identify items as either “old” or “new” when unsure (see Eq. 3). Neutral, optimal bias is reflected in a *c* of 0, with increasing tendencies to respond “old” and “new” reflected in increasingly negative and positive *c* values respectively. Given the dissociation of recognition performance into two measures under single item recognition tasks, set against the summarisation of the NOR task into one measure, there exists the possibility that *DR* may reflect the contribution of both *d*′ and *c*, or that it may reflect only one, or even neither of them.

2 $$d^{\prime} =z(H)-z(FA)$$

3 $$c=-\,\frac{z(H)+z(FA)}{2}$$

Presentation and interpretation of the NOR task in the literature assumes that the *DR* is a measure of recognition memory sensitivity alone. Animals who fail to present a novelty preference are considered to lack the ability to discriminate between old and new items in memory. That is, they are assumed to have low recognition sensitivity. Furthermore, similar to a forced choice task in which participants identify the old item from an array of two items, the NOR task is traditionally considered to be criterion- or bias-free^5^. This assumption is made because animals can perform the task by identifying the newest item presented based on relative memory strength, rather than unnecessarily classifying each item according to an old/new criterion. However, unlike a forced choice task, which typically records only the final memory classification made by the observer, exploration data such as the ratio of time spent exploring old and new items may provide a more nuanced view of the observer’s recognition memory, including their bias. Indeed, the NOR task relies upon an animal’s innate predisposition towards exploring novelty, a reliance that suggests bias may indeed play a role in determining the final metric.

Data from lesion and neurophysiological studies using NOR tasks would suggest that the *DR* is predominantly related to memory sensitivity. Memory sensitivity is unambiguously dependent on structures such as the medial temporal lobes (MTL) and surrounding rhinal cortex^6^, while bias recruits higher order cortical structures such as the prefrontal cortex (PFC) and the parietal cortex^7–9^. Lesions of the perirhinal cortex reliably lead to NOR task performance deficit^10–12^, while PFC lesions do not^11,13^, although PFC lesions have been demonstrated to affect object-place and temporal order NOR variants^14^. However, PFC lesions have been demonstrated to cause novelty orienting difficulties in other tasks^15^. Furthermore, lesions of the posterior parietal cortex leave performance on a standard NOR undisrupted^16^. The impact of parietal lesions on a standard NOR task are yet to be determined. Thus, empirical evidence aimed at ascertaining how the *DR* obtained in the animal literature relates to the components of recognition memory obtained from human research is lacking, making direct comparison of these two extensive literatures difficult. The current study uses a human version of the rodent NOR task combined with a standard test of recognition memory in the same participants to examine how these measures are related.

The procedure we report here was conducted on human participants. Humans do not show the same active physical exploration of new or interesting aspects of their environment as rats. Consequently, we used a visual-paired comparisons (VPC) task which provided new and old item dwell times used to calculate *DR*. The VPC is analogous to the NOR as it relies upon the human tendency to preferentially fixate upon novel objects under free viewing conditions^17,18^. This novelty preference is innate, the task requires no rule learning, and is also unrewarded^17,18^. As such the VPC is closely analogous to the NOR task used in rodents. It has previously been used in infants^18^, older adults with cognitive impairments^19^ and non-human primates^20^ and is well suited to minimising demand characteristics, necessary when translating behavioural tasks across human and non-human species.

Manns, Stark & Squire^21^ provide the only explicit examination of the VPC as a measure of declarative memory in humans to date. Surprisingly, the mean proportion of time spent looking at the novel item in the VPC was uncorrelated to the proportion of those same items correctly identified during a subsequent SIR recognition task. Importantly, the use of the VPC as an encoding task for the subsequent SIR poses problems for interpretation of these results. As discussed by the authors, longer dwell times (or the sum of fixation durations) for a novel item in the VPC task inevitably result in shorter dwell times for the old item paired with it, as both items cannot be viewed simultaneously. As such, based on the assumption that longer dwell times reflect deeper encoding, stronger novelty preferences, or biases, may inevitably lead to decreases in the correct identification of old items paired next to a novel item, as these have not been fixated upon much during the VPC. This is supported by the finding in the same experiment that *DR* correlated with both reaction times and participants’ confidence, which are commonly reported to be highly related to recognition memory ability^22,23^. If hits and correct rejections were separately measured, rather than overall accuracy, this could have been investigated by the authors, highlighting the importance of considering both of these aspects of recognition memory sensitivity separately. This study also leaves the separable components of sensitivity and bias and their relationships to the *DR* unconsidered.

We used two recognition tasks, a VPC task, yielding dwell-time-based *DR*, and a SIR task, yielding response-based sensitivity (*d*′) and bias (*c*) parameters, to interrogate associations between the metrics in the same participants. To vary the depth of encoding, and thus the magnitude of the memory signals recovered within the SIR task^24^, we had participants encode items using shallow and deep encoding manipulations. Potential relationships between the measures (a single *DR* and two sets of memory-strength-dependent *d*′ and *c* parameters) are explored. Given the assumptions of the literature reviewed above, we hypothesised that *DR* would be positively correlated with sensitivity but display no correlation with bias. We anticipated that this selective relationship would hold across both encoding conditions as we have no reason to assume that this relationship exists only at higher or lower memory strengths.

## Results

### Visual Paired Comparison Task: Discrimination Ratio (DR)

**Figure 1:**
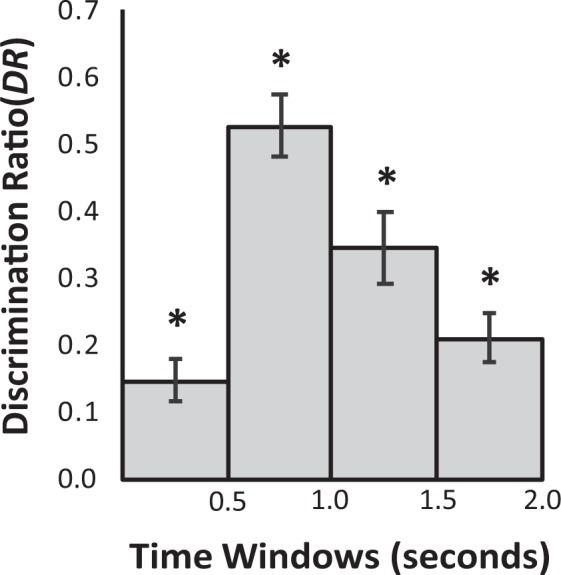
*DR* for each 0.5 second subdivision of the VPC. Error bars represent standard errors of the mean.

We included only data from trials where participants fixated on both study items and both test items (84.91% of all trials). A Wilcoxon signed-rank test confirmed that the left/right position of the new item at test did not significantly affect the included percentage of trials based on the above criteria (left: *M* = 83.39% *SD* = 16.10; right: *M* = 86.43%, *SD* = 13.87), *Z*(n = 28) = −1.34, *p* = 0.180. As assessed using a left/right-determined *DR* (M = −0.017, SD = 0.105) compared to a test value of 0, dwell times were not significantly different for the two identical study items presented in each study trial, *t*(df = 27, n = 28) = −0.84, *p* = 0.410, *d* = 0.16. Importantly for the validity of the VPC task, the mean old/new-determined *DR* (M = 0.321, SD = 0.15) was significantly greater than 0, indicating that participants’ fixations were directed more towards new than old items during test trials, *t*(df = 27, n = 28) = 11.26, *p* < 0.001, *d* = 2.13. To examine the time course of the novelty related response the 2 s VPC response window was split into 0.5 s subdivisions. While *DR* was significantly greater than chance throughout the 2 s response window (Fig. 1, all *t*s > 4.58, *p*s < 0.001, *d*s > 0.87, n = 28) there was a significant difference between the 0.5 s subdivisions, with *DR* starting low, increasing dramatically between 0.5–1 s and then declining in the final second, as outlined by a repeated measures ANOVA with a Greenhouse-Geisser correction, *F*(df = [2.31, 62.48], n = 28) = 17.26, *p* < 0.001, *η*^2^_*p*_ = 0.390 (Fig. 1). Post-hoc Bonferroni corrected pairwise comparisons identified that significant differences were only present between the *DR* for 0.5–1.0 and all other time windows (*DR* 0.5–1.0 [M = 0.528] vs 0.0–0.5 [M = 0.148], *p* < 0.001, *d* = 1.19; *DR* 0.5–1.0 vs 1.0–1.5 [M = 0.346], *p* = 0.007, *d* = 0.68; *DR* 0.5–1.0 vs 1.5–2.0 [M = 0.211], *p* < 0.001, *d* = 1.14). No differences were observed between the other time windows (all mean differences < 0.197, all *ps* > 0.056).

### Single Item Recognition Task: Sensitivity (d′) and Bias (c)

**Figure 2:**
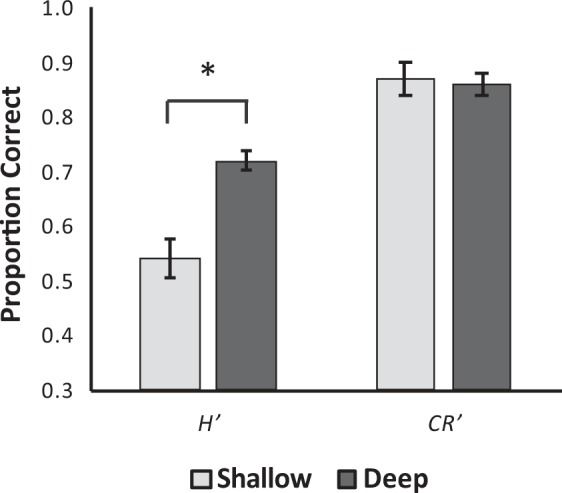
Mean adjusted hit (H′) and correct rejection (CR′) rates according to encoding condition. Error bars represent standard errors of the mean.

Adjusted hit (*H*′) and correct rejection (*CR*′) rates are used to calculate sensitivity and bias (see Methods). As such, we first present analyses of these measures, before examining sensitivity and bias directly. Figure 2 presents mean *H*′ and *CR*′ under conditions eliciting either shallow or deep levels of processing, and thus encoding (see Methods). A Wilcoxon signed-rank tests demonstrated that *CR*′ rates were unaffected by encoding condition (shallow: *M* = 0.873, *SD* = 0.09; deep: *M* = 0.862, *SD* = 0.11), *Z*(n = 28) = −0.66, *p* = 0.508, while *H*′ rates were lower under shallow than deep encoding as outlined by a paired-samples *t*-test (shallow: *M* = 0.541, *SD* = 0.19; deep: *M* = 0.716, *SD* = 0.17), *t*(df = 27, n = 28) = 5.57, *p* < 0.001, *d* = 1.05. These results indicate that the encoding depth of processing manipulation was successful in strengthening the memory traces of those items encoded under a deep level of processing.

**Figure 3:**
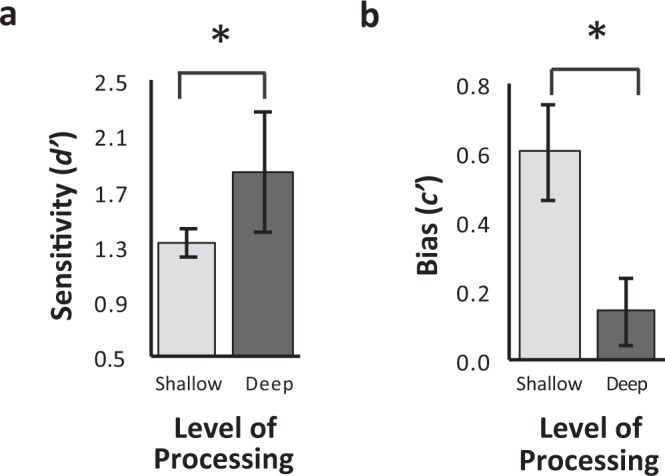
Mean a. sensitivity and b. bias estimates according to encoding condition. Error bars represent standard errors of the mean.

Mean *d*′s for each encoding condition are presented in Fig. 3a. As would be expected based on the *H*′ differences, participants had significantly lower *d*′ in the shallow than the deep condition as outlined by a paired-samples *t*-test (shallow: *M* = 1.34, *SD* = 0.55; deep: *M* = 1.84, *SD* = 0.76), *t*(df = 27, n = 28) = 4.28, *p* < 0.001, *d* = 0.81. To account for variation in *d*′ across levels of processing, *c* was scaled to *d*′ on a participant-by-participant basis to give a measure of relative bias (*c*′; see Methods). Mean *c*′ for each level of processing are presented in Fig. 3b. Participants had a significantly lower *c*′ in the shallow than the deep condition (shallow: *M* = 0.60, *SD* = 0.73; deep: *M* = 0.14, *SD* = 0.54), as outlined by a Wilcoxon signed-rank test *Z*(n = 28) = −3.83, *p* < 0.001. That is, shallow levels of encoding led to more conservative criterion placements, resulting in a decreased tendency to identify stimuli as “old”.

### Associations between recognition memory measures

**Figure 4:**
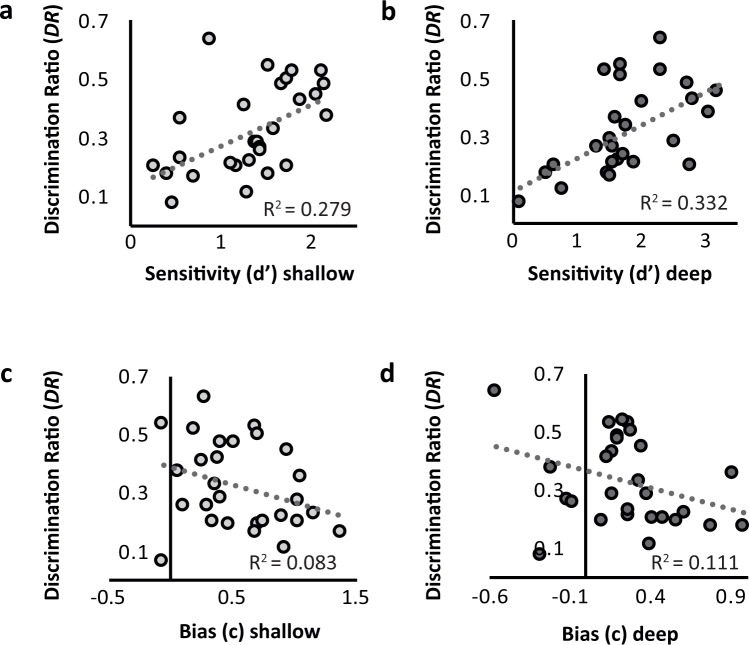
Scatterplots showing DR and d′ for a. shallowly encoded stimuli, and b. deeply encoded stimuli, and between *DR* and *c* for c. shallowly encoded stimuli, and d. deeply encoded stimuli.

The analyses below were critical in motivating this study. We hypothesised that there would be a significant positive relationship between *DR* and sensitivity, in the form of *d*′, but not bias, in the form of *c*. Pearson’s correlations supported these hypotheses. Significant positive correlations were observed between *DR* and *d*′ for both the shallow, *r*(df = 26, n = 28) = 0.528, *p* = 0.004, and deep, *r*(df = 26, n = 28) = 0.576, *p* = 0.001, encoding conditions (Fig. 4a,b). No correlations were observed between *DR* and *c* in either shallow, *r*(df = 26, n = 28) = −0.297 *p* = 0.125, or deep conditions, *r*(df = 26, n = 28) = −0.325 *p* = 0.092, (Fig. 4c,d). (Unsurprisingly, these relationships were maintained in two multiple regression analyses, one using shallow parameters as predictors and another using deep parameters as predictors. In both regressions, *d*′ [*p*s < 0.008] but not *c* [*p*s > 0.154] emerged as a significant predictor of *DR* in significant models [regression model *p*s < 0.009].) These analyses suggest that *DR* does indeed specifically reflect recognition memory sensitivity, but appears unrelated to recognition memory bias.

Using frequentist statistics allows testing of the alternative hypothesis, however it does not allow quantification of the evidence for the null hypothesis. Therefore it is unknown whether the lack of a relationship between *DR* and *c* reflects the true lack of a relationship between these or whether the data is inconclusive. As such, we also subject the data to Bayesian analyses, which do allow such quantification. Similarly to the above correlations, Bayesian correlation tests were run between *DR* and *d*′ and *DR* and *c*. For these, no relevant background knowledge was inputted into the model, and as such Beta priors were set to 1. For correlations with *d*′ the alternative hypothesis of a positive correlation was tested, while for correlation with *c* the null hypothesis of no correlation was tested. The Bayesian correlation test demonstrate the same observed correlation between *DR* and shallow and deep *d*′s as the above Pearson’s correlations, *r* = 0.528 (95% CI: 0.179, 0.735) and *r* = 0.576 (95% CI: 0.238, 0.765) respectively, with associated Bayes Factors of 24.63 and 62.16 respectively. This highlights that the alternative hypothesis of a positive correlation between *DR* and *d*′ predicts the shallow data set 24.63 times better than the null hypothesis, and predicts the deep data set 62.16 times better than the null hypothesis. Bayes Factor robustness checks for different Beta prior widths (0.0–2.0) were also performed. The above results were considered to be robust as Bayes Factors did not vary significantly with different Beta prior widths above 0.2. All Bayes Factors for Beta prior widths above 0.2 were above 20 (suggesting strong evidence) for the shallow analysis, and above 30 (suggesting very strong evidence) for the deep analysis.

The Bayesian correlation test demonstrate the same observed correlation between *DR* and shallow and deep *c’*s as the above Pearson’s correlations: *r* = −0.297 (95% CI: 0.581, 0.084) and *r* = −0.325 (95% CI: −0.600, 0.056) respectively, with associated Bayes Factors of 1.39 and 1.10 respectively. This highlights that the null hypothesis of no correlation between *DR* and *c* predicts the shallow data set only 1.39 times better than the alternative hypothesis that the data are correlated, and predicts the deep data set only 1.10 times better than the alternative hypothesis that the data are correlated. Bayes Factor robustness checks for different Beta prior widths (0.0–2.0) were also performed. The above results were considered to be anecdotal for all Beta prior widths. These analyses provide further evidence that the result from this experiment show a significant and important relationship between *DR* and *d*′, but that the relationship between *DR* and *c* is inconclusive, as it neither supports the alternative nor the null hypothesis.

**Figure 5:**
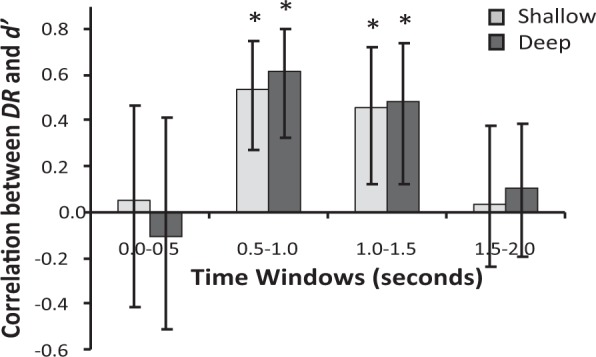
Mean correlation coefficients between *DR* and *d*′ for each 0.5 s window, according to encoding condition. Error bars represent bootstrapped 95% confidence intervals estimated using Monte-Carlo case resampling (with replacement) bootstrapping for 10 000 simulated experiments.

Given that we found *DR* to vary across the 0.5 s time windows (Fig. 1), we also ran separate correlations between the *DR*s from each window and *d*′ according to encoding condition. Mean Pearson’s correlation coefficients for these analyses are presented in Fig. 5. For this figure, 95% confidence intervals were estimated using Monte-Carlo case resampling (with replacement) bootstrapping procedures. The available data set for each time window was sampled with replacement until the total number of participants (n = 28) was reached. This was repeated 10 000 times, simulating 10 000 experiments, and the confidence interval was calculated from this bootstrapped data set. Of the four time windows, *DR* was only significantly positively correlated to shallow and deep *d*′s between 0.5 s and 1.0 s, *r*(df = 26, n = 28) = 0.540, *p* = 0.003 and *r*(df = 26, n = 28) = 0.613, *p* = 0.001 respectively, and between 1.0 s and 1.5 s, *r*(df = 26, n = 28) = 0.458, *p* = 0.014 and *r*(df = 26, n = 28) = 0.484, *p* = 0.009 respectively. There were no significant correlations with either shallow or deep *d*′s in the early 0.0 s to 0.5 s window, *r*(df = 26, n = 28) = 0.051, *p* = 0.759 and *r*(df = 26, n = 28) = −0.108, *p* = 0.583 respectively, or in the late 1.5 s 2.0 s window, *r*(df = 26, n = 28) = 0.039, *p* = 0.844 and *r*(df = 26, n = 28) = 0.106, *p* = 0.590 respectively. This selectivity of association indicates that, even within the narrower time windows which all show significantly positive *DR*s, there are only some windows in which the critical association with sensitivity is present. The associated windows coincide with observed peak *DR*s across the sample, and windows for which the variance in *DR* was greatest (0–0.5 s window: SD = 0.17; 0.5–1.0 s window: SD = 0.25; 1.0–1.5 s window: SD = 0.28; 1.5–2.0 s window: SD = 0.19).

## Discussion

This experiment is the first to provide empirical validation of the *DR* as a measure of recognition memory sensitivity in humans. This has wide reaching implications for tests of human memory and potentially for interpreting the extensive number of experiments using the NOR paradigm for testing memory sensitivity in rodents.

We first demonstrated that participants spent more time visually dwelling upon new compared to old stimuli during a VPC task. Importantly, participants’ preferential orienting to novelty, as summarised by the *DR*, were significantly positively correlated with recognition memory sensitivity but not bias. That is, participants who spent a greater proportion of time dwelling on novel items were also better able to differentiate old from new items in a standard recognition memory task. Notably, these participants were neither more conservative nor liberal in their willingness to identify items as “old”, with this lack of relationship being stronger according to frequentist rather than Bayesian methods. Interestingly, the *DR*-sensitivity correlation was only reliable for *DR*s obtained during the 0.5 s to 1.0 s and 1.0 s to 1.5 s time windows within the entire 2.0 s visual examination period.

The absence of a correlation between *DR* and sensitivity in the 0.0–0.5 and 1.5–2.0 second time windows occurred despite a significant novelty orientation (positive *DR*) in the VPC being observed for all time windows. Thus, this association is not simply driven by the progression of novelty orientating across a trial^25^. Rather, it may be the result of increased variance in *DR* during these windows. This waxing and waning of variance across the time windows suggests that individual differences in the progression of participants’ *DR*s are important to the association. It may be that the time-course of preferential novelty orienting differs across participants. All participants are quick to show this behaviour, which eventually disappears^25^. However, some participants may maintain their novelty preference for most of the duration of the trial before it is attenuated, while others have already stopped or significantly reduced it. Such behaviour patterns would be consistent with the presence of lower *DR*s as well as the reduced variance within these extreme windows. The nature and extent of this individual variability and the effects on the associations with memory sensitivity, remain to be fully investigated, but should be considered when using *DR* as a measure of memory sensitivity. Researchers in this position may wish to select periods of maximal *DR* as best indicative of individual differences in the capacity to differentiate novel from familiar stimuli.

As well as supporting the use of the *DR* as a measure capable of reflecting recognition memory sensitivity, our findings demonstrate that it appears not to be affected by preferences to respond to old or new stimuli, or at a more conservative level that the relationship between *DR* and bias are inconclusive. While it was conceived that the ratio of time spent exploring old and new items may provide a more nuanced view of the observer’s recognition memory than simply recording participants’ final memory classification, and thus may include bias, the dwell-time-based *DR* did not reflect this. Taken together with other sources of evidence, such as the current human literature which regards the VPC as a bias-free forced-choice task^5^, and the lesion and neurophysiological studies demonstrating no effect of PFC^14^ or posterior parietal cortex^16^ lesions on NOR tasks despite its impact on novelty orienting^15^, it is reasonable to suggest the lack of a correlation found using frequentist statistics, and reflected as inconclusive using Bayesian statistics reflects the lack of involvement of bias throughout this task. In essence, within the scope of these different evidence sources, the current data support the suggestion that the *DR* in the current VPC task is bias free.

An important qualifier here is that the retention period over which we spaced VPC study and test phases was 0.5 seconds, which contrasts with longer periods typically used in rodent research (minutes to days). It is possible that these differences could potentially impact the presence or absence of relationships observed in the present study. Drawing on work that has examined SIR bias and SIR sensitivity as a function of long retention intervals (15 minutes to 3 months^26^ we know that long delays reduce both bias and sensitivity. Crucially though, we are not aware of any reported changes in the covariance of these parameters over time. Thus, we would not anticipate changes in the relationships reported here, but the effect of retention interval on the bias-sensitivity relationship remains an empirical question to be explored in future work.

One further important consideration is that design of the VPC and SIR tasks mean they have a number of key differences. For example, the VPC was implemented in such a way as to minimise participants being consciously aware that this was a memory task. This was done deliberately to reflect the NOR task used in rodents as accurately as possible. However, the differences between the tasks could result in differences in the memory mechanisms that support them. One possibility is that memory could be retrieved using recollection in the SIR while the VPC could potentially be solved using familiarity. This could mean that while performance in these tasks is well correlated the underlying neural mechanisms could be different. This does not detract from the current findings which are a crucial first step to understanding how transferable measures of recognition memory are across tasks and species. It does, however, demonstrate the need for further investigation in this area.

The empirical validation of the *DR* as a measure of recognition memory sensitivity has consequences for recognition memory research in non-standard human populations. The VPC is a non-verbal, short (<15 mins) task that requires no instruction to complete. For this reason, it has been frequently used in human infant research^18^. While previous research has demonstrated the appropriateness and value of the VPC within a clinical setting^19,27^, it is not until the results of the current experiment that researchers could have confidence in the aspects of recognition memory being tested by the task. As we have demonstrated here that novelty orientation in the VPC provides a good analogue to individual’s recognition memory sensitivity, we argue that it provides an excellent alternative to SIR tasks for this measurement in those who have impaired ability to follow instructions or coordinate key-presses, such as people with dementia and people undergoing rehabilitation following brain injury.

It is important to note that a correlational approach underpins the conclusions drawn above. Acknowledging the caveats to such an approach, we draw conclusions which refrain from declaring or specifying the nature of the relationship. Indeed, future research will be required to explore whether a single or multiple parallel cognitive processes contribute to each, any or any combination of the *DR*, recognition memory sensitivity or bias. Certainly, such research leading to an understanding of the cognitive and neural substrates contributing to these will specify why the *DR* is such a valid measure of sensitivity, appearing to leave bias unaccounted for.

The current data also have implications for our understanding of how the NOR task, used to assess recognition memory in rodents, can be interpreted. The assumption in the animal literature that the degree of novelty orienting during the standard NOR is an indicator of the sensitivity with which an animal is able to discriminate old from new stimuli has been widely held but never tested. Recent studies have examined whether NOR performance is driven primarily by response to new or old items^28,29^ but these studies still assume that differential responding on the basis of relative novelty represents a measure of memory sensitivity. The current findings crucially verify this assumption in humans, and suggest that the use of the *DR* in animal and comparative recognition memory research to study memory sensitivity is valid. While further empirical testing and validation of these findings with animals is required, the current finding is nonetheless of considerable importance given the scope and dominance of the NOR within the literature, and the number of animals sacrificed to studies dependent upon this task^2^. Where the NOR has been used to evaluate drug effects^3^ and pharmacological compound safety^30^, the current validation of the *DR* provides much needed confidence in the aspects of memory tested. Furthermore, where the NOR task is used to investigate the functions of defined neurological areas^10,11,31^, the validation of the *DR* as a measure of memory sensitivity will enable a more detailed functional anatomy map to be developed/conceptualised. The current contribution towards its validation, along with the versatility of the NOR in rats (which can be tested using visual, olfactory or tactile stimuli)^32^ and the possibility of using the VPC in humans suggests that *the DR* is a highly suitable measure for translational research between species.

The specific correlation of *DR* with sensitivity and not bias highlights a common problem in the description of the how rodents explore novelty. Discussing the behaviour demonstrated by animals in the NOR as a preferential exploration of novel as compared to old items is accurate but can be misleading. The innate novelty preference leading to positive *DR*s should not be confused with a mnemonic bias, or a preference to consider items as new or old under conditions of uncertainty. To avoid confusion, the *DR* should be discussed in terms of a novelty orienting response rather than a “preference” for novel items.

In view of such translational research, one aspect of the current VPC needs consideration. Here the *DR* measure used was calculated as an average from numerous (~34 trials) VPC trials. This differs from the task as it is run with rodents, where *DR* is typically calculated from standard NOR tasks based on a relatively small number of trials (~4 trials, but can be as few as 1). Theoretically this means we are obtaining a *DR* with a greater signal to noise ratio in humans, and therefore a *DR* calculated from a smaller number of trials in rodents may be less related to their recognition memory sensitivity. While this may be the case, it is also important to consider that human participants will have been exposed to a significantly richer visual environment in their life pre-experiment than laboratory rodents will, and as such will have significantly greater interference for the items presented to them on screen (despite efforts to make these as novel as possible). Thus, empirical testing of the number of trials required to obtain a *DR* which is highly and reliably correlated to recognition memory is required in both humans and rodents.

In summary, our findings validate the use of *DR* as a measure of recognition memory sensitivity uncontaminated by bias. They also suggest that, on a sample level, there may be certain windows within the period of exploration that are better representative of sensitivity than others. These windows here coincided with observations of maximal *DR*, but this remains to be fully explored across tasks and species. Finally, we suggest that *DR* may also be a useful measure in human samples, particularly those with verbal or motor difficulties. A small number of studies using the VPC in human samples have shown promise and, based on our current results, we see no reason why this methodology should not be developed and extended to enable memory assessment in a far broader range of samples than are currently served by traditional recognition tests requiring instruction and response.

## Methods

All testing procedures were approved by and comply with the University of St Andrews University Teaching and Research Ethics Committee (UTREC; approval code: PS11888).

### Participants

Thirty-seven participants with self-reported normal or corrected-to-normal vision were tested. They were compensated £7 for their time. Nine participants were excluded from the analyses for the following reasons: failure to follow task instructions (1); self-reported estimate that >35% of stimuli were familiar or recognized from pre-experimental exposure (3); poor eye movement calibration (>0.5 degree average spatial error) (1); failure to fixate on both stimuli presented on screen during the visual paired comparison task on more than 50% of trials (3); failure to exceed chance performance in the SIR task (*d*′ < 0.1) (1). The final sample consisted of 28 participants (75.68% of the original sample; 20 females; mean age = 23.86 years, age range = 18–32 years). Informed consent was obtained in accordance with the University Teaching and Research Ethics Committee of St Andrews.

### Stimuli

A set of 432 colour Pokémon generation II-VI (© 1995–2016 Nintendo/Creatures Inc./GAME FREAK inc. Pokémon) and Digimon (© 1997–2008 Bandai) characters were selected from online databases (“Pokémon Wiki”, n.d.; “Wikimon”, 2005). Images measured 200 × 200 pixels. For each participant, a set of 320 items was randomly sampled from this pool, 80 of which were used for the Visual Paired Comparison (VPC) task, 240 for the single item recognition (SIR) task.

### Apparatus

#### Visual paired comparison task (VPC)

Eye movements were recorded using an SR Eyelink 1000 eye tracker (SR Research Ltd., Mississauga, Ontario, Canada) with tower mount apparatus, sampling at 250 Hz. Fixations and saccades were determined using a displacement threshold of 0.1 deg spatial resolution, a velocity threshold of 30°/s and an acceleration threshold of 8000°/s^2^ (SR Research Ltd, 2013). Participants were seated in front of a CRT computer screen, resolution 1280 × 1024, used to display the stimuli. A chin rest placed 40 cm from the screen reduced participants’ head movements and increased participants’ comfort. Eye movements were calibrated using a nine-point calibration, and ensured that recordings had a mean spatial error <0.5 deg for each participant. During calibration, participants were asked to fixate on the fixation crosses presented individually on screen. Drift corrections to confirm that calibrations were still valid were also implemented throughout the experiment (see Procedure section below). Again, a mean spatial error <0.5 deg was required for validation, where a mean spatial error >0.5 deg triggers re-calibration using a nine-point calibration. The task was run using Matlab (The Mathworks Inc., Natick, MA, R2011b) and Psychophysics Toolbox^33^.

#### Single item recognition task (SIR)

The SIR task was run on a PC laptop, with a screen resolution of 1024 × 768 pixels, using Matlab (The Mathworks Inc., Natick, MA, R2011b) and Psychophysics Toolbox^33^. Responses were made by keypress and presentation was self-paced by the participant, where the mean presentation/response times in seconds were as follows: study items in the shallow blocks M = 1.27, SD = 0.38; study items in the deep blocks M = 1.68, SD = 0.59; test items in the shallow blocks M = 1.26, SD = 0.37; test items in the deep blocks M = 1.22, SD = 0.33.

### Procedure

All participants undertook the VPC task, immediately followed by the old/new image recognition task to avoid priming participants to consider the VPC task as a memory task.

#### VPC task

**Figure 6:**
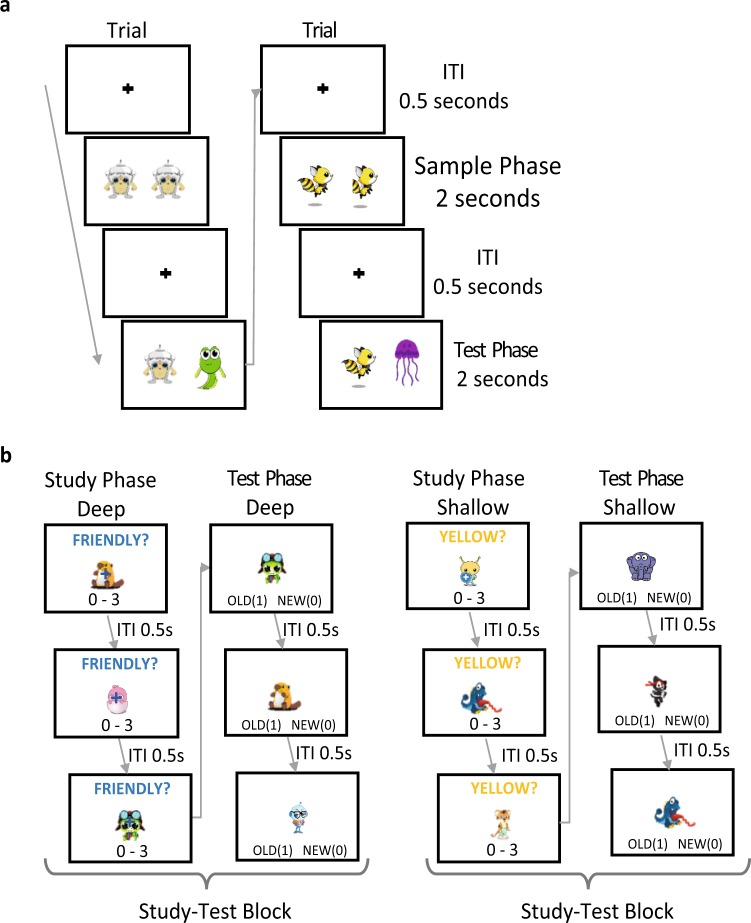
Methodology schematic. (**a**) Example of two trials during the VPC. (**b**) Example of three study trials and three test trials for both the shallow and the deep level of encoding blocks. ITI denotes inter-trial-intervals. Images presented here are placeholders and not from the set of Digimon and Pokemon stimuli used in the experiment for copyright purposes. All images used in this figure were obtained from www.pixabay.com and are available under Creative Commons (CC0 1.0 Universal).

After an initial calibration, participants were presented with a series of 40 trials. Each trial consisted of a sample phase lasting 2 seconds, during which two instances of a novel item were presented for the first time, and a test phase also lasting 2 seconds in which one instance of the previously presented, now old item was presented alongside a novel item (Fig. 6a). The two items presented at study and test were displayed side-by-side (inter-stimulus gap of 158 pixels) on a white screen for 2 seconds. Participants were instructed to “simply look at the screen as if you are watching TV”. No overt recognition responses were required of participants. Lures were presented with equal probability on the left and right hand sides of the screen.

Each phase was presented for 2 s with a 0.5 s inter-trial interval, during which a black fixation-cross appeared centrally against a white background onscreen. Breaks were offered every 10 trials, during which participants had the opportunity to remove their heads from the chin-rest. Eye tracking calibrations were undertaken after every 10 trials, regardless of whether participants had taken the break. Drift corrections were also implemented every 5 trials to ensure adequate eye tracking accuracy. After completion of the 40 trials, participants were seated at a separate table in the same testing room and completed the SIR task.

#### SIR task

Participants completed two self-paced study-test blocks, each consisting of a study phase followed by a test phase. Each study phase consisted of a series of 60 serially presented study items displayed centrally onscreen with a 0.5 s inter-trial interval between study items, consisting of a black fixation-cross appearing centrally against a white background onscreen. Participants completed an incidental encoding task with different levels of processing in a blocked manner. The order of the two study-test blocks, and therefore the level of processing used at study, was counterbalanced between participants. A question displayed at the top of the screen indicated whether participants were being asked to rate the amount of yellow on an item (“YELLOW?”, from “0” = “none” to “3” = “lots”; shallow processing) or rate the items friendliness (“FRIENDLY?”, from “0” = “very unfriendly” to “3” = “extremely friendly”; deep processing) for the given block (Fig. 6b). Encoding questions (“YELLOW?” in yellow, “FRIENDLY?” in blue) remained onscreen throughout the response period.

Each test phase consisted of a series of 120 serially presented test items displayed centrally onscreen. Sixty test items were targets (items seen during the preceding study phase) and 60 were lures (novel items not previously been seen at study), presented in a random order. There was a 0.5 s inter-trial interval between test items, consisting of a black fixation-cross appearing centrally against a white background onscreen. For each item, participants made self-paced “OLD” or “NEW” judgements, responding by key press (1 = “OLD”, 0 = “NEW”).

## Calculations

Mirroring the measures used in the animal NOR literature, a dwell-time-based Discrimination Ratio (*DR*), measured as a proportional dwell time, was the principal measure obtained from the visual paired comparison task. The *DR* reflects the preferential exploration allocated to a novel item as compared to a familiar one, as a proportion of total exploration time (to control for intrinsic variability in participants’ levels of exploration). Here, dwell times were used as a proxy for exploration time. For a given Test trial, fixation durations for the lure were summed (to give a lure dwell time, *T*_*new*_) as were those for the target item (to give a target dwell time, *T*_*old*_). The sum of all fixation durations for both items was used as the total trial exploration time (*T*_*total*_). The *DR* was calculated as outlined in Eq. 1.

For sample phases, we also calculated a left/right preference *DR*. Here dwell times for novel and familiar items are substituted by dwell times for the left and right items, as outlined in Eq. 4.

4 $$D{R}_{left/right}=\,\frac{{T}_{(left)}-\,{T}_{(right)}}{{T}_{(total)}}$$

Overall performance on the Image Judgement Task was investigated using sensitivity (*d*′) and bias (*c*) parameter estimates from the equal variance signal-detection model^5^. Hit and False Alarm rates were adjusted for errorless responding as per^34^. These adjusted hit (*H*′) and false alarm (*FA*′) rates were calculated as outlined in Eqs 5 and 6, based on the absolute numbers of hits (*h*), misses (*m*), correct rejections (*cr*) and false alarms (*fa*).

5 $$H^{\prime} =\,\frac{h+0.5}{h+m+1}$$

6 $$FA^{\prime} =\,\frac{fa+0.5}{fa+cr+1}$$

The adjusted correct rejection rate, *CR*′, is simply 1 - *FA*′. Sensitivity (*d*′) and bias (*c*) were calculated as in Eqs 2 and 3, substituting *H* for *H*′ and *CR* for *CR*′. When there are differences in *d*′ the same absolute *c* can reflect different levels of bias (relative to a participant′s sensitivity). Under these circumstances, *c* can be divided by *d*′, yielding *c*′^5^.

### Statistical analysis

All statistical tests were two-tailed. An α threshold of 0.05 was adopted for all frequentist statistical analyses reported. Normality of data sets were established using Shapiro-Wilks tests, and are presented in Table 1 below. It is acknowledged that measures Shapiro-Wilks test sensitivity and specificity are limited with the current sample sizes^35^, however, we would expect the power of these to be broadly comparable to the values of 0.51 reported from simulations run by for similar sample sizes^35^. Where data was not normally distributed, a non-parametric test appropriate to the data being analysed was selected as detailed below.

**Table 1:** Shapiro-Wilk test for normality.

Variable	*W*	df	*p*
Number new items presented on the left of the screen	0.863	28	0.002*
Number new items presented on the right of the screen	0.863	28	0.002*
*DR* for study items	0.986	28	0.959
*DR* for test items	0.950	28	0.203
*DR* for test items between 0.0–0.5 s	0.931	28	0.066
*DR* for test items between 0.5–1.0 s	0.938	28	0.099
*DR* for test items between 1.0–1.5 s	0.970	28	0.593
*DR* for test items between 1.5–2.0 s	0.953	28	0.23
*H*′ shallow	0.958	28	0.313
*H*′ deep	0.943	28	0.133
*CR*′ shallow	0.831	28	<0.001*
*CR*′ *deep*	0.775	28	<0.001*
*d*′ shallow	0.947	28	0.170
*d*′ deep	0.959	28	0.326
*c* shallow	0.968	28	0.527
*c* deep	0.966	28	0.486
*c*′ *shallow*	0.740	28	<0.001*
*c*′ deep	0.684	28	<0.001*

Results for Shapiro-Wilks normality test undertaken on all variables, including degrees of freedom and p values.

Note: *denotes significance at an α = 0.05 level, and thus suggests non-normally distributed data.

Absence of a Left/Right *DR* in VPC study trials was analysed using a Wilcoxon signed-rank test as the data violated the assumption of normality (see Table 1). Novelty preference (i.e. the presence of a positive *DR*) for VPC test trials was analysed using a one-sample *t*-test. Time course of the *DR* was analysed using a repeated measures ANOVA, with Greenhouse-Geisser correction, followed by Bonferroni corrected pairwise post hoc comparisons. The effect of level of encoding on adjusted Hit rates was analysed using paired-samples *t*-test, and on adjusted Correct Rejections using a Wilcoxon signed-rank test as data for Correct Rejections were not normally distributed (see Table 1). The effect of level of encoding on sensitivity (*d*′) and was analysed using a paired-samples *t*-test, and on adjusted bias (*c*′) using a Wilcoxon signed-rank test as data for Correct Rejections were not normally distributed (see Table 1). The relationships between *DR* and both sensitivity (*d*′) and bias (*c*′) were analysed in a frequentist manner using Pearson’s correlation, and furthered by running multiple regressions. These same relationships were also analysed using Bayesian correlation tests. These were run using JASP (JASP Team, 2018, v0.8.6), where default parameters were used and run with a Beta prior of 1. Model robustness was tested across priors between 0 and 2 through the JASP interface.

### Data availability

The dataset generated and analysed during the current study are available from the corresponding authors on reasonable request.
